# Effects of *Chlorella* extracts on growth of *Capsicum annuum* L. seedlings

**DOI:** 10.1038/s41598-022-19846-6

**Published:** 2022-09-14

**Authors:** Shi-Lin Tian, Abid Khan, Wen-Na Zheng, Li Song, Jun-He Liu, Xiao-Qian Wang, Li Li

**Affiliations:** 1 School of Biological Science and Food Engineering, Huanghuai University, Zhumadian, 463000 Henan People’s Republic of China; 2 Department of Horticulture, The University of Haripur, Haripur, 22620 Pakistan

**Keywords:** Plant sciences, Agroecology

## Abstract

The long-term application of chemical fertilizers has caused to the farmland soil compaction, water pollution, and reduced the quality of vegetable to some extent. So, its become a trend in agriculture to find new bio-fertilizers. *Chlorella* extract is rich in amino acids, peptides, nucleic acids, growth hormones, potassium, calcium, magnesium, iron, zinc ions, vitamin E, B1, B2, C, B6, folic acid, free biotin and chlorophyll. *Chlorella* extract can promote biological growth, mainly by stimulating the speed of cell division, thereby accelerating the proliferation rate of cells and playing a role in promoting plant growth. Whether *Chlorella* extract can be used to improve the growth of pepper (*Capsicum annuum*), needs to be verified. In current study, a pepper variety 'Chao Tian Jiao' was used as experiment material, by determining the changes of the related characteristics after spraying the seedlings with *Chlorella* extract, and its effect on growth of *Capsicum annuum* plants was investigated. The results showed that the *Chlorella* extract significantly increased plant height of pepper seedlings (treatment: 32.2 ± 0.3 cm; control: 24.2 ± 0.2 cm), stem diameter (treatment: 0.57 ± 0.02 cm; control: 0.41 ± 0.03 cm) and leaf area (treatment: 189.6 ± 3.2 cm^2^; control: 145.8 ± 2.5 cm^2^). Particularly, the pepper seedlings treated with *Chlorella* extract, developed the root system in better way, significantly increased the chlorophyll *a*, and the activities of SOD, POD and CAT enzymes were also improved significantly. Based on our results, we can speculate that it is possible to improve the growth of *Capsicum annuum* seedlings and reduce the application of chemical fertilizers in pepper production by using *Chlorella* extract.

## Introduction

Pepper is a one-year or limited perennial herb, an important agricultural crop due to the nutritional value of its fruits, and an excellent source of a wide array of phytochemicals with well-known antioxidant properties (carotenoids, capsaicinoids, phenolic compounds, particularly flavonoids, quercetin, and luteolin), which is known as the king of vitamin C in vegetables^1,2^. The pepper is very popular among those who enjoy spicy foods, and its cultivated area in China is growing year after year^3^.

In recent decades, the use of chemical fertilizers has increased crop yield, however, the long-term application of chemical fertilizers has caused the soil aggregate structure destroyed, causing soil compaction and soil beneficial bacteria death, and the nitrate content of the vegetables exceeded the standard^4,5^. Therefore, reducing chemical fertilizer and increasing crop productivity have become the main research direction of agricultural production in China.

One approach to increasing crop productivity is the development of environment-friendly bio-fertilizers. To increase the content of nutritional constituents in plants, many approaches have been studied such as genetic selection, which has included allele selection, gene and genome duplication, and new genotypes creation. However, despite the advantages that these techniques offer, some of them may also pose potential problems for food safety and require special attention in order to ensure consumer health protection^6,7^. On this account, the use of bio-fertilizers in agricultural practices is proposed as a safe tool to enhance the nutritional properties of food crops. Bio-fertilizers are recognized as environment-friendly compounds with beneficial effects on plants^8,9^. In particular, they decrease the use of chemical fertilizers by increasing the amount of micro- and macro-nutrients taken up by plants, positively influencing root morphology and plant growth^10,11^. They also display hormone-like activity and influence plant metabolism by interacting with biochemical processes and physiological mechanisms^12–14^.

Recent studies suggest that active molecules contained in bio-fertilizers can promote nitrogen assimilation^8–12^. Furthermore, the induction of the metabolic pathway associated with the synthesis of phenylpropanoids in plants treated with bio-fertilizers may explain why these products can help plants to overcome stress situations^15,16^. In recent studies, the application of products with bio-fertilizers action to pepper plants was found to exert positive effects on plant growth^14,17,18^. Therefore, searching for bio-fertilizers has recently become a trend in agricultural production.

*Chlorella* is a single-cell green algae organism, that contains a large amount of various functional compounds such as crude protein 50–60%, carbohydrate 15–20%, crude lipid 12–18%, growth hormones, potassium, calcium, magnesium, iron, zinc, vitamin E, B1, B2, C, B6, folic acid, free biotin and chlorophyll^19,20^. *Chlorella* has high photosynthetic efficiency, which is the only plant on the Earth that can grow four times in 20 h, and it is known as the ‘canned sun’. Now, *Chlorella* extract has been used as bio-fertilizer in many crops, like Chinese chives, spinach^19^, lettuce^21^, wheat^22^ and *Hibiscus esculentus*^23^, which can increase the biomass of the above crops.

At present, the application of *Chlorella* extract in pepper plants has not been seen. To examine whether *Chlorella* extract can stimulate the growth of pepper seedlings as a bio-fertilizer, further research is needed. The purpose of this study is to investigate the effects of *Chlorella* extract as bio-fertilizer on the growth of pepper seedlings.

## Results

### Effect of Chlorella extract on plant height of pepper seedlings

**Figure 1:**
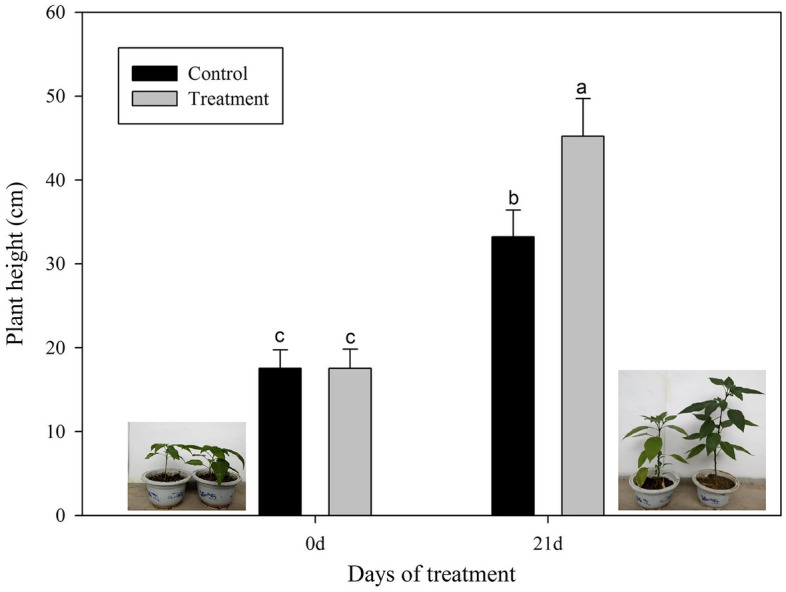
Effects of *Chlorella* extracts on the plant height of pepper. Plant height was measured on the 0 and 21th day after *Chlorella* extract treatment. Control: no *Chlorella* treatment; Treatment: plants were treated by *Chlorella* extracts; SAS analysis at 5% is shown in lowercase letters to indicate significant differences. Vertical bars in the figures represent ± SD of five independent biological replicates.

The leaf surface of the pepper was sprayed with *Chlorella* extract, and the growth status of plants were analyzed at 0, 7th, 14th and 21st day post treatment. After 21 days of *Chlorella* extract treatment, the plant height of the *Chlorella* extract-treated plants were significantly higher than that of the control pepper plants (Fig. 1).

### Effects of Chlorella extract on the stem diameter, leaf area and fruit growth of pepper

**Figure 2:**
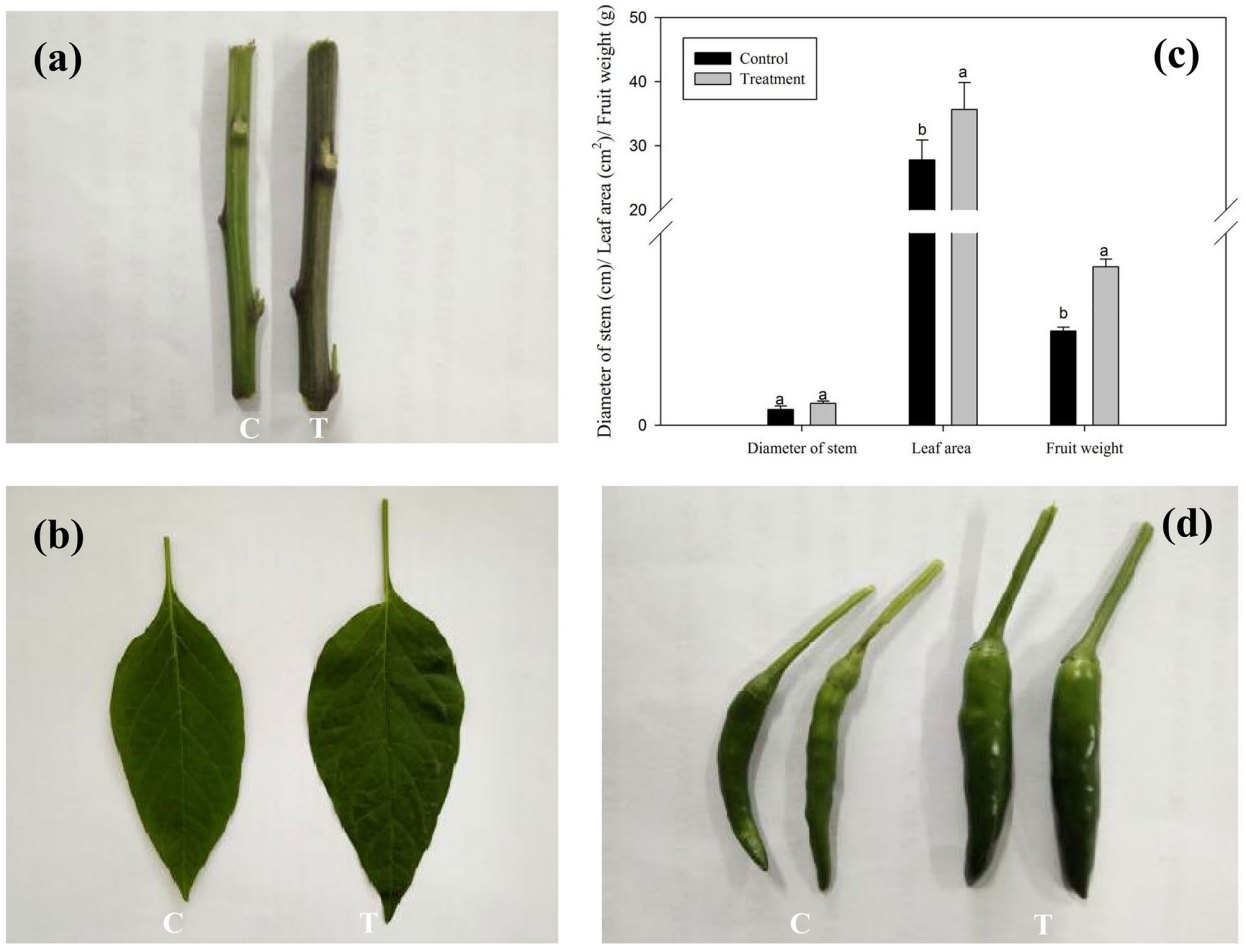
Effects of *Chlorella* extracts on the stem diameter, leaf area and fruit growth of pepper. The stem diameter, leaf area and fruit weight were measured on the 21th day after *Chlorella* extracts treatment. Control: no *Chlorella* extracts treatment; Treatment: plants were treated by *Chlorella* extracts; “C” means “Control group”, and “T” means “Treatment group”. SAS analysis at 5% is shown in lowercase letters to indicate significant differences. Vertical bars in the figures represent ± SD of five independent biological replicates.

**Figure 3:**
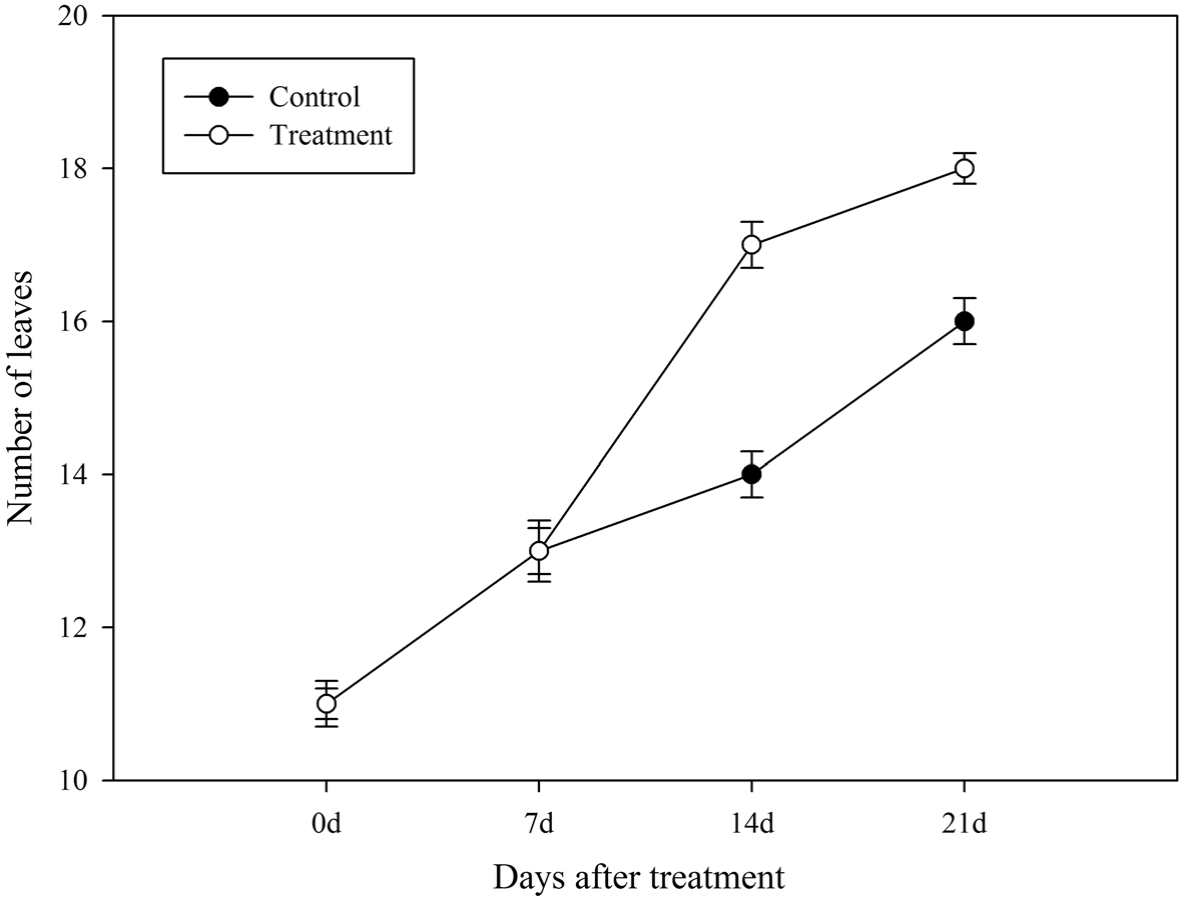
Effects of *Chlorella* extracts on number of leaves plant^-1^. The number of leaves plant^-1^ (Control group and Treatment group) were counted from the 0 to 21th day after *Chlorella* extracts treatment. Control: no *Chlorella* extracts treatment; Treatment: plants were treated by *Chlorella* extracts; SAS analysis at 5% is shown in lowercase letters to indicate significant differences. Vertical bars in the figures represent ± SD of five independent biological replicates.

After 21 days of treatment with *Chlorella* extract, the stem diameter, leaf area and fruits growth of the control and treated plants were measured. The stem diameter of the treated plants were slightly thicker than the control (treatment: 0.57 ± 0.02 cm; control: 0.41 ± 0.03 cm), while leaf area (treatment: 189.6 ± 3.2 cm^2^; control: 145.8 ± 2.5 cm^2^) and fruits weight (treatment: 5.12 ± 0.02 g; control: 3.74 ± 0.05 g) of the treated plants were significantly increased as compared to the control plants (Fig. 2). *Chlorella* extract post-treatment, not only the leaf area of the pepper plants increased (Fig. 2b and c), but number of leaves of the pepper plant also increased significantly (Fig. 3). On 14th and 21st day post *Chlorella* extract treatment, the number of leaves plant^-1^ was much higher than the control group (Fig. 3).

### Effect of Chlorella extract on the chlorophyll content of pepper plants

**Figure 4:**
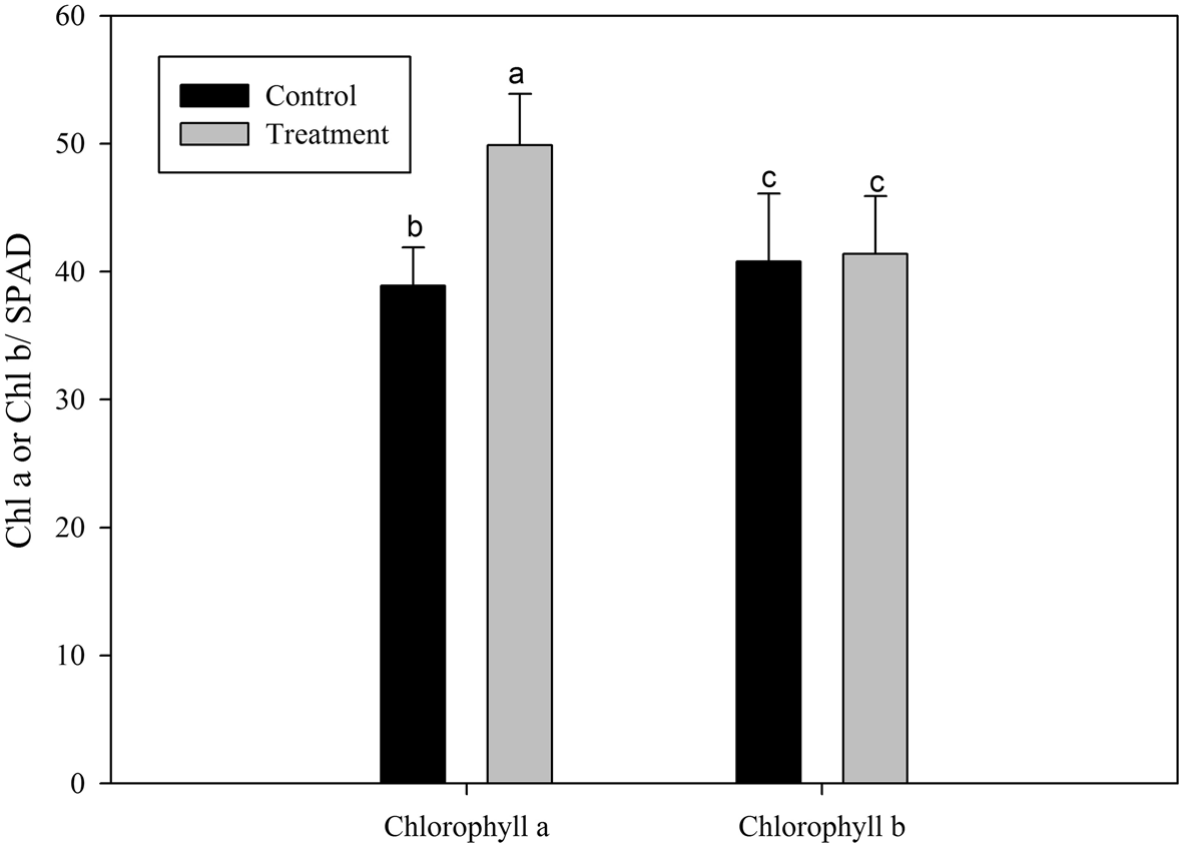
Effect of *Chlorella* extracts on the Chlorophyll content of pepper plants. Chlorophyll *a* and chlorophyll *b* were measured on 21st day after *Chlorella* extracts treatment. Control: no *Chlorella* extracts treatment; Treatment: plants were treated with *Chlorella* extracts; SAS analysis at 5% is shown in lowercase letters to indicate significant differences. Vertical bars in the figures represent were presented as mean ± SD, all experiments were carried out in triplicate.

Data regarding *Chlorella* extract effect on the chlorophyll content of pepper plants leaves is shown in Fig. 4. After 21 days of *Chlorella* extract treatment, the SPAD value of chlorophyll *a* and chlorophyll *b* were measured in the treated and the control group. It was noted that chlorophyll *a* was increased significantly in the *Chlorella* extract treated plants, while chlorophyll *b* did not; indicating that *Chlorella* extract has a greater impact on chlorophyll *a* (Fig. 4).

### Effect of Chlorella extract on root growth of pepper

**Figure 5:**
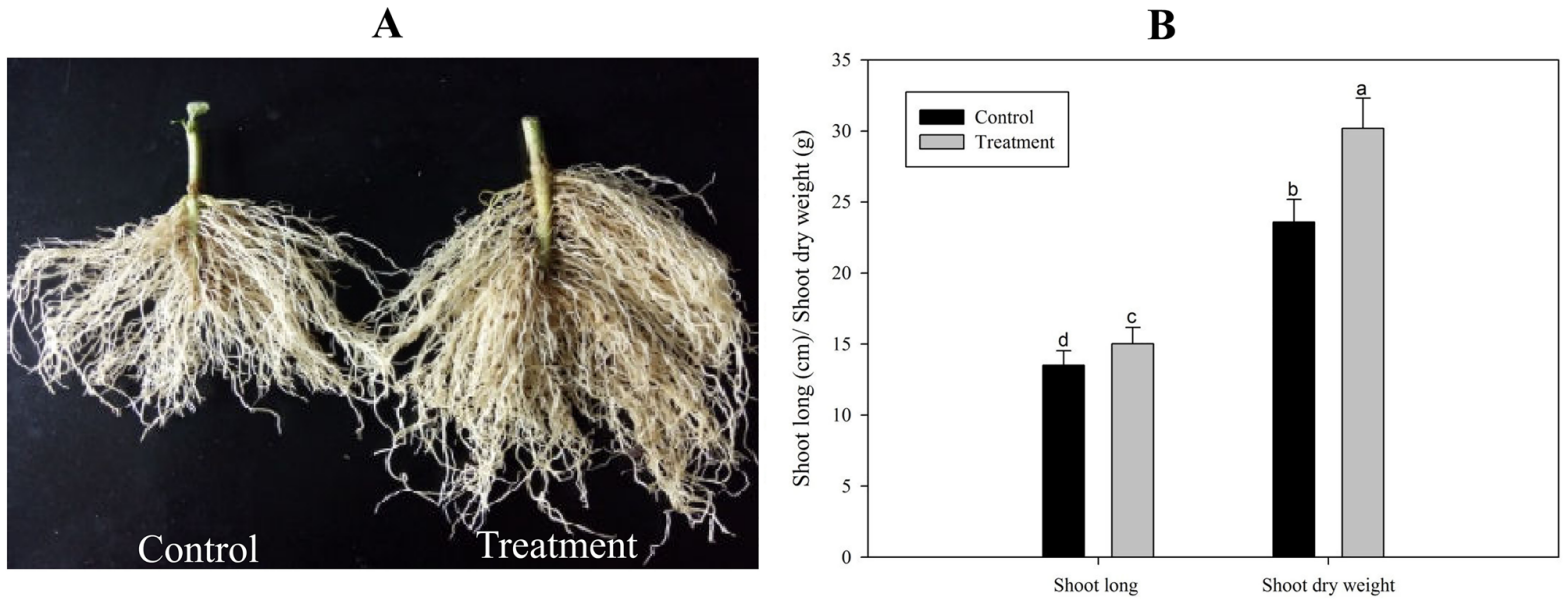
Effect of *Chlorella* extracts on the growth of pepper roots. Shoot length and shoot dry weight of the pepper plants (Control group and Treatment group) were measured on 21th day after *Chlorella* extracts treatment. Control: no *Chlorella* extracts treatment; Treatment: plants were treated by *Chlorella* extracts; SAS analysis at 5% is shown in lowercase letters to indicate significant differences. Vertical bars in the figures represent ± SD of five independent biological replicates.

In order to investigate the effect of *Chlorella* extract on root growth characteristics of pepper plants, the plants were pulled out after 21 days of spraying with *Chlorella* extract, the soil was washed away, and the root growth of the treated and the untreated control plants was compared. The results showed that there was a significant difference in the root growth of the treated and control plants (Fig. 5). Specifically, the fibrous roots of treated plants were larger, denser and the root length as well as dry weight were significantly increased as compared to the untreated control plants (Fig. 5).

### Changes of activity of SOD, POD and CAT enzymes

**Figure 6:**
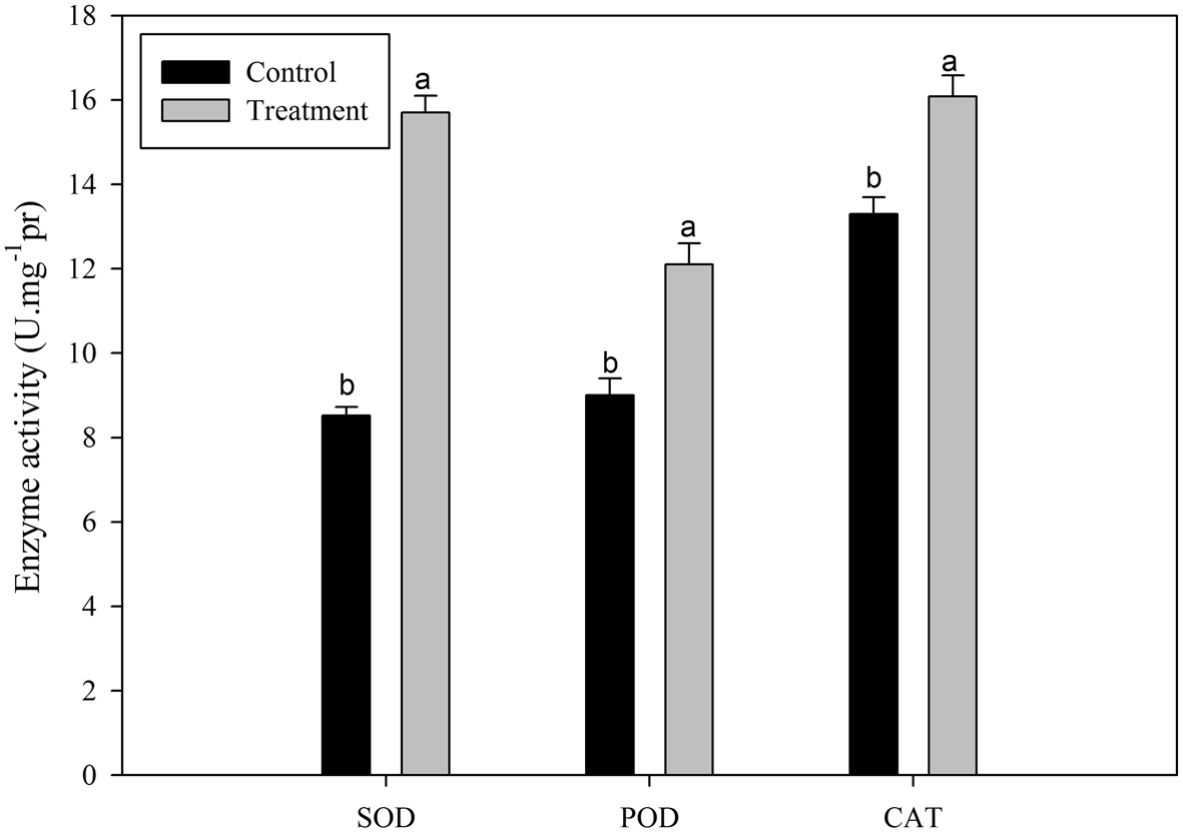
Changes in related enzyme activities. Activities of the SOD, POD and CAT enzymes in pepper leaves (Control group and Treatment group) were measured on 21st day after *Chlorella* extracts treatment. Control: no *Chlorella* extracts treatment; Treatment: plants were treated by *Chlorella* extracts; SAS analysis at 5% is shown in lowercase letters to indicate significant differences. Vertical bars in the figures represent ± SD of five independent biological replicates.

Data reported in Fig. 6. show that *Chlorella* extract significantly increased the activity of SOD (control 8.5 ± 0.2 U.mg^-1^.pr; treatment 14.6 ± 0.4 U.mg^-1^.pr), POD (control 9.0 ± 0.4 U.mg^-1^.pr; treatment 14.1 ± 0.5 U.mg^-1^.pr), and CAT (control 16.0 ± 0.4 U.mg^-1^.pr; treatment 22.0 ± 0.5 U.mg^-1^.pr). By and large, compared to the control, the activity of SOD, POD and CAT enzymes in the leaves of *Chlorella* extract treated peppers were significantly higher (Fig. 6).

## Discussion

*Chlorella* extract has been used as bio-fertilizer in many crops, like Chinese chives, spinach^19^, lettuce^19^, wheat^22^ and Hibiscus esculentus^23^, which can increase the biomass of the above crops. The average height of Chinese chives treated with the *Chlorella* was 3.7 cm smaller than that of the untreated. The leaf width and fresh weight of Chinese chives treated with the chlorella was 0.5 mm wider and 30.3 g heavier than that of the untreated. The commercialization and yield of Chinese chives treated with the chlorella was 11.9% and 18.3%, respectively higher than that of the untreated. The thickness and number of spinach leaves treated with chlorella was 27.9% and 41.8%, respectively higher than that of the untreated. The fresh weight and yield of the spinach treated with the chlorella was 63.6% and 31.5%, respectively higher than that of the untreated^19^. The combined seed and soil inoculation of *Chlorella vulgaris* speed up growing of the Hibiscus esculentus, increase pod yield^23^. A recent study on wheat discussed about the importance of *Chlorella* extract in stimulating plant growth^26^. It was found that as compared to control, the *Chlorella* extracts increased the wheat plant height by 30%, and total dry matter biomass of the above- and below-ground parts were increased by 22% and 51%, respectively^26^. Sugar beet (*Beta vulgaris* subsp. vulgaris) is a commercially important biennial root crop, providing about 20% of the world’s annual sugar production. *Chlorella* extracts had a positive effect on sugar beet germination by increasing efficiency and regularity of this critical process for *B. vulgaris* seeds. Further studies have shown that *Chlorella* extracts contain cytokinin^24,27^, that could be the possible reason for increase in photosynthetic biomass^28^. The effect of the application of *Chlorella* extracts, foliar spray and root drenching, was evaluated in lettuce seedlings by monitoring their morpho-biometric parameters and chlorophyll, carotenoid, and total protein contents. The results show that *Chlorella* extract positively affected the growth of lettuce seedlings, increasing the dry matter, chlorophyll, carotenoid, and protein contents in the edible portion of the plant^22^.

In our research, we found that *Chlorella* extract positively affected the morphological characteristics (such as plant height, stem diameter, total number of leaves) and the chlorophyll content in pepper leaves; as well as significantly improved the root development. Secondly, by spraying *Chlorella* extract, the pepper seedlings could keep a well-developed root system (Fig. 5) and the related antioxidant enzymes activity were also improved (Fig. 6).

Some researchers found that the influence of *Chlorella* extract on cell metabolisms was discovered to be mostly due to the physiological action of major and minor nutrients, amino acids, vitamins, and plant growth regulators on cellular metabolism in treated plants, resulting in increased growth and crop yield^24,25^.

The photosynthesis of *Chlorella* is very strong, which is dozens of times that of other plants. The main reason why *Chlorella* can grow rapidly is that it contains *Chlorella* Growth Factor (CGF), which is quite rich in nucleoprotein, Nucleic acid, ribonucleic acid (RNA), deoxyribonucleic acid (DNA), various vitamins, amino acids, polysaccharides, complex protein bodies, enzymes, glycoproteins, various plant hormones, etc., it may be the reason for its ability to promote plant growth.

## Conclusion

*Chlorella* extract significantly increased plant height of pepper seedlings, stem diameter and leaf area. Particularly, pepper seedlings treated with *Chlorella* extracts, its root system was developed in better way, chlorophyll *a* increased significantly, and the activities of SOD, POD and CAT enzymes were also improved significantly. Based on the above results that *Chlorella* extracts contributes to the growth and development of pepper plants, we can conclude that with *Chlorella* extracts treatment it is possible to reduce the application of chemical fertilizers in pepper production.

## Methods

### Materials

Plant materials *Capsicum annuum conoides* ‘Chao Tian Jiao’ and *Chlorella vulgaris* strain were used in the experiment, and the cultural operations were performed according to the relevant guidelines^29^.

The plant material *Capsicum annuum conoides* 'Chao Tian Jiao' was provided by the innovation team of pepper core germplasm resources creation and molecular breeding of Henan province while the *Chlorella* strain was provided by the Zhumadian International Joint Laboratory of Pepper Modern Breeding.

### Chlorella cultivation

The *Chlorella vulgaris* was separately cultivated in Bold Basal culture medium (BBM) according to the method of Kim et al.^30^ Briefly, the strain of *Chlorella vulgaris* was placed in a control room at constant temperature (30 °C) for 6 days in a shaking incubator at the speed of 180 rpm, while the pH of the culture medium was 6.0. After seven days, the *Chlorella* was harvested when its dry biomass concentration was about 180 g/L in 7.5 L bench scale fermenter^29^.

### Preparation of Chlorella extracts

500 g of fresh *Chlorella* was separately immersed (w/v; 1:10) in 5000 mL ethanol for 72 h, and stirred from time to time. The process was repeated until the extracted solvent became colorless. The *Chlorella* extracts were filtered separately through a medium flow filter paper (Whatman 40,8 μm). The extract was concentrated in vacuo using a rotary evaporator at 50 °C while lyophilizing the extract. The condensed ethanol extract was dried at ambient temperature before being placed in an airtight amber vial and kept at 4 °C in a desiccator. While using the extract, it was first solubilized with dimethyl sulfoxide (DMSO) and then *Chlorella* extracts were diluted with distilled water to a concentration of 0.4 mg/L^30^. The leaves of pepper seedlings were sprayed with extracts solution, whereas the untreated control plants were sprayed with distilled water. Each treatment consists of five plants, and each plant was sprayed with 100 mL solution of *Chlorella* extracts. The nozzle was about 10 cm away from the pepper leaves during spraying.

### Pepper seedlings cultivation

The experiments were carried out in greenhouse. Seeds were sown in a cold frame, and seedlings were transplanted to the pots (Pot specifications: 20 cm × 14 cm, Color: White) when it had developed 8–10 true leaves, the growth medium of these pots: sterile mixture of peat: sand: perlite (1:1:1); Hoagland solution was used to provide nutrients for potted pepper growth. There were 10 replicates for each treatment, one per pot, and the pots were arranged randomly in the greenhouse. Two weeks later, the seedlings of treatment groups were sprayed with the *Chlorella* extracts at 18:00 on the same day, whereas the mock control plants were sprayed with distilled water instead of *Chlorella* extracts^19^. Plants were sprayed with *Chlorella* extracts, the amount of *Chlorella* extracts were 100 mL per time, once per 10 min, and three times in total.

### Determining the relevant indicators of plant morphology

The height of the pepper plants were measured with a measuring tape, the stem diameter was measured using vernier calipers, and the leaf area was measured by a number grid method. The shape of the leaf is drawn on a transparent coordinate sheet, and then the grids are counted. When calculating the grid, the blade edge is calculated as 1 if it exceeds half a grid; and the blade edge will be not counted if it is less than half grid. The leaf area is calculated based on the number of grids counted. All above-mentioned indexes were measured three times, and the results are expressed as mean ± standard deviation^31^.

### Sample collection

Pepper leaves from the control and *Chlorella* extracts treated plants were collected on 0th day and 21st day separately, placed in ice boxes, and immediately taken to the laboratory for assay.

### Determination of chlorophyll content

SPAD value of chlorophyll *a* and chlorophyll *b* were measured by a chlorophyll analyzer (SPAD502 Plus Chlorophyll Meter, Spectrum Technologies, Inc.); each sample was measured three times and their average value was calculated^32^.

### Determination of enzyme activities

Superoxide dismutase (SOD), peroxidase (POD) and catalase (CAT) activities of the control and treated plants leaves were measured by nitrogen blue tetrazolium (NBT) method, spectrophotometry and ultraviolet absorption^33^. All the specific activities of the enzyme fractions were calculated based on the amount of protein in the fraction^34^.

### Data processing

SAS 6.12 software (SAS Institute, Gary, North Carolina) was used for data analysis. All measured values were presented as mean ± standard deviation of the means. Duncan’s multiple-range test was chosen, and least significant ranges (LSR) analysis at 5% significantly differences were shown; and figures were drawn using Sigma Plot 10.0.
